# A vexing case of a 73‐year‐old man with fevers, orbital cellulitis, and asymptomatic interstitial lung disease

**DOI:** 10.1002/rcr2.70020

**Published:** 2024-09-08

**Authors:** Sushil Agwan, Lai‐Ying Zhang, Thomas Baker, Michael Lane, David Godbolt, John A. Mackintosh

**Affiliations:** ^1^ Department of Thoracic Medicine The Prince Charles Hospital Chermside Queensland Australia; ^2^ Faculty of Medicine The University of Queensland Herston Queensland Australia; ^3^ Department of Clinical Immunology and Allergy Royal Brisbane and Women's Hospital Herston Queensland Australia; ^4^ Anatomical Pathology The Prince Charles Hospital Chermside Queensland Australia

**Keywords:** interstitial lung disease, orbital cellulitis, VEXAS

## Abstract

VEXAS (Vacuoles, E1 enzyme, X‐linked, Autoinflammatory, Somatic) syndrome is a rare and recently identified disease resulting from a somatic mutation in the X‐linked UBA1 gene in cells of myeloid lineage. It can present in a myriad of ways with the potential to affect various organ systems, including the lungs. VEXAS is usually steroid responsive, but no strong data exists for the use of a steroid‐sparing agent. There is limited emerging evidence for haematopoietic stem cell transplantation in a select number of cases. Regardless, prognosis for this condition is poor and a treatment algorithm remains a priority. Herein, we present a case of VEXAS that came to attention with discovery of a relatively asymptomatic interstitial lung disease and led to recurrent febrile episodes with evolving multi‐organ involvement.

## Graphical abstract

We present a unique case of VEXAS that came to attention with discovery of a relatively asymptomatic interstitial lung disease and led to recurrent febrile episodes with evolving multi‐organ involvement.

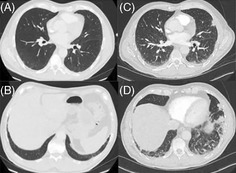

## INTRODUCTION

Vacuoles, E1 enzyme, X‐linked, Autoinflammatory, Somatic (VEXAS) syndrome is a rare and only recently recognized disease associated with a somatic mutation in the X‐linked *UBA1* gene in myeloid‐lineage cells^1^, ^2^ resulting in reduced protein ubiquitination due to overproduction of a dysfunctional form of the ubiquitin‐activating enzyme, E1. Ubiquitination is crucial for targeted protein degradation^3^ and disruption of this process results in activation of the innate immune system, excess production of inflammatory cytokines such as interleukin‐6, interleukin‐8, tumour necrosis factor, and interferon‐gamma by activated T and B cells, and enrichment for macrophage and neutrophil activity^4^ with resultant autoinflammation and cellular stress. Although case studies of this disease are still relatively limited, the most common mutation observed in the literature is p.Met41 with the most frequently documented missense substitutions being to threonine (p.Met41Thr), valine (p.Met41Val), and leucine (p.Met41Leu).^4^, ^5^

VEXAS can present in a myriad of ways with respiratory involvement being a common phenomenon. Herein, we present a perplexing and unique case of VEXAS which came to attention through the finding of asymptomatic interstitial lung disease and led to recurrent presentations to hospital with fevers and multi‐organ involvement.

## CASE REPORT

**FIGURE 1:**
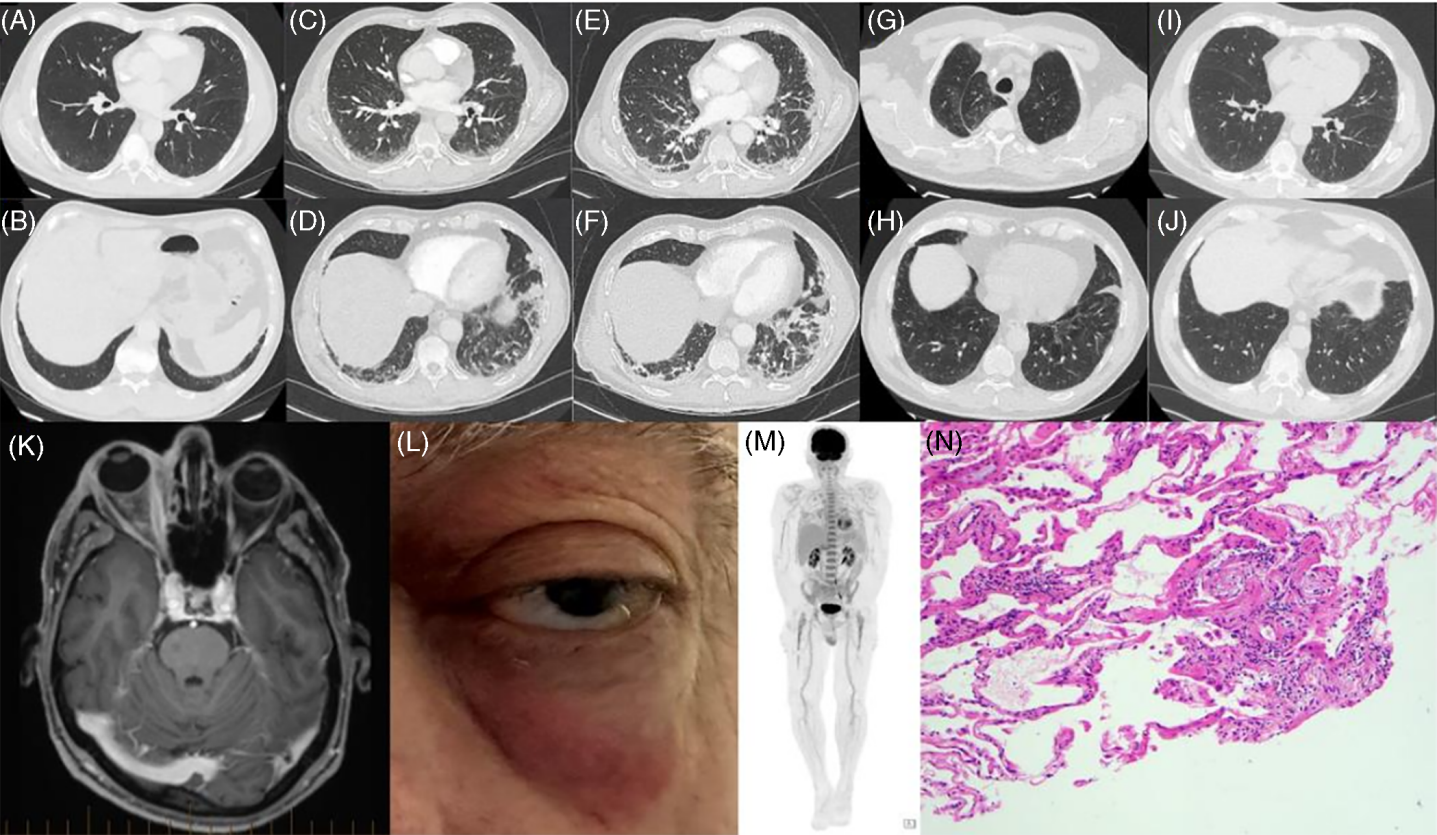
(A and B) Initial chest computed tomography performed contemporaneously with presentation for right orbital swelling, demonstrating subtle subpleural ground glass opacity; (C and D) chest computed tomography performed during presentation with pleuritic chest pain, 6 weeks following scans A and B, demonstrating progressive ground glass opacity and consolidation consistent with non‐specific interstitial pneumonitis and organizing pneumonia radiological pattern; (E and F) follow‐up chest computed tomography 6 weeks after scans C and D, demonstrating significant progression of consolidation; (G and H) follow‐up chest computed tomography after commencement of mycophenolate, demonstrating improvement in consolidation, but persistent ground glass opacity; (I and J) follow‐up chest computed tomography demonstrating near complete resolution of ground glass opacity following treatment with ruxolitinib. (K) MRI demonstrating right peri‐orbital swelling. (L) Re‐presentation with contra‐lateral periorbital swelling in the left eye. (M) Positron emitted tomography scan demonstrating moderate FDG avidity within the bone marrow. (N) Lung cryobiopsy specimen (×100 H&E) demonstrating focal minimal organizing pneumonia with some apparent incorporation into the interstitium, with a focal minimal interstitial lymphoid infiltrate.

A 73‐year‐old male presented to the respiratory clinic for investigation of incidental findings from a computed tomography pulmonary angiogram (CTPA) performed to evaluate self‐resolving pleuritic chest pain (Figure 1C,D). It showed patchy mid and lower zone ground‐glass opacities in both a central and peripheral distribution, as well as patchy bilateral peripheral consolidation associated with limited traction of the airways. Radiologically, this was suggestive of an overlapping non‐specific interstitial pneumonitis (NSIP) and organizing pneumonia (OP)‐like pattern, therefore hinting towards an active, inflammatory ILD rather than a fibrotic sub‐type. The patient's background included dyslipidaemia and hypertension with no smoking or relevant occupational/environmental exposures identified. At this stage he was largely asymptomatic, and thus, planned for a short‐term follow‐up with a repeat chest CT.

A few weeks earlier, he had presented with fevers, night sweats, and relapsing–remitting right eye pain with swelling, and lacrimation, along with new unprovoked upper and lower limb superficial venous thromboses. Magnetic resonance imaging (MRI) of the eyes (Figure 1K) revealed orbital inflammation. A chest CT at that time had shown subtle ground glass opacity (Figure 1A,B). This episode was treated with a prolonged course of weaning oral prednisolone after failure of empirical antibiotics for suspected periorbital cellulitis.

At clinic follow‐up, 6 weeks after the initial respiratory review, a repeat CT chest showed progressive inflammatory parenchymal lung changes with increasing consolidation and ground glass opacity (Figure 1E,F). Now with oral prednisolone weaned to 5 mg/day, he had recurrence of fevers followed shortly by development of left temporal pain, unilateral orchitis, dysphagia and oral ulcers and, the development of left orbital swelling (Figure 1L). Patient's blood count revealed mild normocytic anaemia (115 g/L, normal 120–180 g/L) and lymphopenia (0.85 × 10^9^/L, normal 1.4–4.0 × 10^9^/L). C‐reactive protein (CRP) levels were consistently elevated (>100 mg/L) over months. An extensive infective and auto‐immune screen was unremarkable while a transthoracic echocardiogram did not demonstrate any valvular abnormalities. Lung function testing demonstrated normal spirometry (FEV1 2.66L, 96% predicted), FVC (3.46L, 95% predicted) and mild reduction in DLCOc (16.40 mL/min*mmHg, 72% predicted). A positron‐emitted tomography scan (Figure 1M) showed moderate, diffusely increased fluorodeoxyglucose activity throughout the axial and proximal appendicular bone marrow. Subsequently, a transbronchial cryo‐biopsy (Figure 1N), targeting the lower lobe abnormalities, was undertaken and the histology identified scattered foci of organizing pneumonia, but no evidence of vasculitis, capillaritis, or granulomata. With no clear cause identified for the patient's progressive ILD and evolving multi‐system involvement, he ultimately underwent a bone marrow trephine and aspirate, which confirmed Met41Val variant of the UBA1 gene and mild neutrophil vacuolation. This confirmed a diagnosis of VEXAS, thus explaining the myriad of inflammatory organ involvement (including the lungs, as demonstrated by the ground glass and consolidative changes) that the patient developed over a short period of time. Subsequently, his prednisolone dose was promptly escalated to 25 mg/day and mycophenolate was commenced as a steroid sparing agent. However, despite a concomitant mycophenolate dose of 1 g twice daily, he was unable to wean the prednisolone dose below 15 mg/day without symptom recurrence and persistence of the ground glass infiltrate on CT (Figure 1G,H). Therefore, his steroid sparing agent was switched to ruxolitinib. Following this he was able to successfully reduce the prednisolone to 7.5 mg/day. Follow‐up chest CT showed complete resolution of the ground glass opacity suggesting responsiveness to ruxolitinib (Figure 1I,J).

## DISCUSSION

Clinically, VEXAS syndrome presents in adulthood with a predisposition for older males. Characteristic features include constitutional symptoms such as recurrent fevers and weight loss, arthralgia, skin lesions, haematological derangement, pulmonary abnormalities, ocular symptoms, venous thrombosis, and chondritis.^6^ It is not uncommon for various components of the VEXAS syndrome to either be misdiagnosed, or to meet criteria for another rheumatological or immunological disease. A recent study performing whole exome sequencing on a prospective cohort of patients diagnosed with relapsing polychondritis in the United States found that up to 8% had an underlying diagnosis of VEXAS.^7^ Histopathology of skin lesions in VEXAS may present as neutrophilic dermatoses and can be mistakenly diagnosed as Sweet's syndrome,^8^ or alternatively may show leukocytoclastic vasculitis mimicking polyarteritis nodosa or giant cell arteritis.^6^ While episcleritis is reportedly the most frequent ocular symptom, periorbital oedema can also be present (as was in this case) and may be misdiagnosed as orbital cellulitis.^6^ Haematologic abnormalities such as macrocytosis and cytopenias, especially thrombocytopenia, are frequently observed, with a French multicentre case series of 116 VEXAS patients noting that 50% of their patients met criteria for myelodysplastic syndrome (MDS) and/or monoclonal gammopathy of unclear significance (MGUS); increased autoinflammation and endothelial dysfunction also predisposes patients to hypercoagulability, resulting in recurrent venous thromboses.^6^ Respiratory symptoms such as dyspnoea and cough are commonly reported, with parenchymal infiltrates of ground glass change or consolidation more frequently observed rather than established pulmonary fibrosis^5^, ^9^; rare cases of airway stenosis secondary to bronchial chondritis are also reported in the literature.^10^ It is therefore important to consider VEXAS syndrome as a rare, but important, differential in systemic inflammatory conditions affecting the typical end‐organs mentioned above, especially if macrocytic anaemia and thrombocytopenia are also present. While diagnosis of VEXAS requires demonstration of a *UBA1* mutation, bone marrow biopsy can reveal the typical cytoplasmic vacuolisation in myeloid and erythroid cell precursors which, in combination with appropriate clinical features, may provide the impetus for more definitive genetic testing.

First‐line treatment for VEXAS remains glucocorticoid therapy, with both methylprednisolone and oral prednisolone having been utilized in the literature with prompt symptom resolution.^2^, ^11^ Unfortunately, most patients are unlikely to tolerate corticosteroid weaning and continue to require >20 mg/day for disease control; the optimal steroid‐sparing agent is still undetermined. While several traditional DMARD (disease‐modifying anti‐rheumatic drug) therapies have been trialled, including methotrexate, mycophenolate, calcineurin inhibitors, and cyclophosphamide, response to many of these agents appear to be transient and patients are usually unable to become entirely corticosteroid‐free.^2^ Tocilizumab, a monoclonal antibody against the IL‐6 receptor, has demonstrated some success in various studies in reducing both cutaneous and systemic inflammation,^12^ as have the JAK (Janus kinase)‐inhibitors ruxolitinib and tofacitinib^13^ Azacytidine has been trialled with some success in VEXAS patients meeting criteria for MDS.^13^ Case series describing allogeneic haematopoietic stem cell transplant (HSCT) in VEXAS‐MDS or VEXAS‐myelofibrosis have described the potential for clinical remission, although study numbers are extremely small.^14^

Currently, VEXAS syndrome remains a rare and frequently under‐recognized cause of multisystem inflammation. Given the recency of its discovery, prognosis is still unclear, but 3‐to‐5‐year mortality rates have been estimated at anywhere between 15% and 50%.^2^ It is evident that current treatment options remain inadequate for long‐term disease control. Further research into this rare condition—including better characterization of its heterogenous clinical manifestations, improved understanding of culprit molecular pathways, and clinical trials to better guide an overarching treatment algorithm—remains a priority.

## AUTHOR CONTRIBUTIONS

Sushil Agwan, Thomas Baker and Lai‐Ying Zhang wrote the manuscript. All authors contributed to editing of the manuscript and approved the final version of the manuscript.

## CONFLICT OF INTEREST STATEMENT

None declared.

## ETHICS STATEMENT

The authors declare that appropriate written informed consent was obtained for the publication of this manuscript and accompanying images.

## Data Availability

Data sharing is not applicable to this article as no new data were created or analyzed in this study.
